# Mechanical characteristics and antibacterial activity against *Staphylococcus aureus* of sustainable cellulosic paper coated with Ag and Cu modified ZnO nanoparticles

**DOI:** 10.1038/s41598-024-79265-7

**Published:** 2024-11-29

**Authors:** Ramadan A. Geioushy, Samya El-Sherbiny, Eslam T. Mohamed, Osama A. Fouad, Marwa Samir

**Affiliations:** 1 Central Metallurgical Research and Development Institute, Helwan, P.O. Box: 87, Cairo, 11421 Egypt; 2 Paper and Printing Laboratory, Chemistry Department, Faculty of Science, Helwan University, Helwan, Egypt; 3 Botany and Microbiology Department, Faculty of Science, Helwan University, Helwan, Egypt

**Keywords:** Antimicrobial effect, Coated paper, Mechanical characteristics, Optical characteristics, Paper ageing, Chemistry, Materials science, Nanoscience and technology

## Abstract

In this study, zinc oxide (ZnO) nanoparticles were prepared and modified using a wet chemical method with different concentrations of Ag and Cu nanoparticles. The objective was to improve the mechanical, optical, and antibacterial properties of the coated paper by using the prepared pigments. The long-term antimicrobial effects of the coated paper were evaluated over 25 years. The successful synthesis of a hexagonal structure of ZnO nanoparticles decorated with spherical Ag and Cu nanoparticles ranging from 20 to 50 nm was confirmed using X-ray photoelectron spectroscopy (XPS), X-ray diffraction (XRD), and transmission electron microscopy (TEM). By increasing the concentrations of Ag and Cu from 0.01% to 1.0%, the mechanical properties of the coated paper were enhanced. The tensile strength reached a maximum of 6.77 kN/m and 7.03 kN/m, elongation increased to 1.69% and 1.70%, tensile energy absorption improved to approximately 77 and 80 J/m^2^, and burst strength rose to 218 and 219 kPa, respectively. The use of Ag-modified ZnO maintains the optical properties, while Cu-modified ZnO reduces brightness and whiteness without affecting opacity. The antimicrobial inhibition activity was improved with higher silver (Ag) and copper (Cu) content. The formulations containing 1% Ag/ZnO and 1%Cu/ZnO showed long-lasting antibacterial effects against gram-positive *Staphylococcus aureus* bacteria. Even after 25 years of aging, they maintained inhibition rates of 92.2% and 62.2%, respectively. The molecular docking and GeneMANIA analysis revealed the potential of ZnO, Ag-modified ZnO, and Cu-modified ZnO nanoparticles to disrupt the *S. aureus* cell wall biosynthesis pathway by targeting the MurA enzyme and associated cell wall synthesis genes.

## Introduction

Nowadays, the whole world has faced increased illnesses caused by bacteria and viruses^1–3^. The coronavirus pandemic of COVID-19 had a great impact on people’s lives, industrial production, and global supply chains. Indeed, increased outbreaks of viral and bacterial infections pose severe challenges to the economy and society^4^. The paper sector plays a crucial role in the worldwide economy^5,6^. Paper serves as a fundamental means of communication, education, artistic expression, packaging, international documentation, and financial exchange^7–9^. Despite its vital role in our lives, contaminated paper facilities the transmission of viral infection from one person to another. It is crucial to protect our health from infection by incorporating an antibacterial layer into the paper used in various aspects of life^10^ and preserving or enhancing the other paper properties. To address this challenge, paper coating can be used afterwards in the paper-making process to fill cavities, cover fibers, incorporating either inorganic or organic antimicrobial compounds into paper surface, and improve the paper’s qualities. Paper coatings typically consist of water-dispersed mixtures containing pigments, binders, and specialized additives. Pigments play a fundamental effect in regulating the characteristics of the coating components^11,12^. It is well known that nanoparticles such as Ag, Cu, TiO_2_, ZnO, CuO, and AgO are efficient antibacterial agents^4,13–19^. Either titanium dioxide (TiO_2_) or zinc oxide (ZnO) are employed as white pigments in paper coatings to enhance the optical and printing characteristics. Zinc oxide (ZnO) possesses distinctive electrical and optical characteristics, making it highly versatile for various applications such as photocatalysis, optical devices, sensors, and semiconductors in solar cells^20–22^. In this regard, the antimicrobial effectiveness of ZnO could be enhanced by incorporating a highly effective antibacterial agent. The broad antibacterial spectrum of Ag and Cu nanoparticles has increased their potential for use in a variety of applications over the past decade. According to our earlier research, it has been established that the application of low-content Ag nanoparticles effectively releases reactive oxygen species and stimulates the production of free radicals, leading to damage to the cell wall of bacteria^4^. However, there is a significant gap in research regarding the synergistic effects of combining nanoparticles such as Ag, Cu, and ZnO to enhance both the antibacterial and optical/mechanical properties of coated paper. Additionally, there is a need to study the long-term retention of these antimicrobial properties.

In this study, we will explore how adding Ag and/or Cu nanoparticles to the surface of ZnO NPs can enhance its antibacterial properties. Ag and Cu-modified ZnO nano pigments could be utilized as coating materials for paper to provide antibacterial activity, preventing the spread of bacterial and viral infections and improving the optical and mechanical properties^23^. Furthermore, the long-term antimicrobial properties of coated paper have been studied for up to 25 years, extending its potential applications to the biomedical and food industries as packaging materials for preserving food. The functional paper coating developed in this study supports several United Nations Sustainable Development Goals (UNSDGs), including Good Health and Well-Being, Industry, Innovation, and Infrastructure, Responsible Consumption and Production, and Climate Action.

## Experimental

### Materials

The provided materials were utilized to prepare Ag/ZnO and Cu/ZnO-modified nanopigments: Zinc acetate (Zn(CH_3_CO_2_)_2_·2H_2_O, purchased from Merck 99.99% trace metals basis), zinc chloride (ZnCl_2_, 98%), and copper (II) chloride dihydrate (CuCl_2_.2H_2_O, 99%) from Sigma Aldrich. Silver nitrate (AgNO_3_, 99%) was bought from Dop Organic Kimya Company. Sodium dodecylsulfate (SDS, 98.5%), hydroxyl ammonium hydrochloride (NH_2_OH.HCl, 99%), ethanol (EtOH, C_2_H_5_OH, 95%), and ammonium hydroxide (NH_4_OH, 25%) were supplied by Adwic Company. Polyethene glycol (PEG, Mwt = 8000) from MP Biomedicals Company was utilized as the reducing agent. Polyvinylpyrrolidone (PVP K 15, Mol wt = 10,000) supplied by Fluka Company was used as a stabilizing agent. The coating mixture was prepared using a common Acronal S 360D binder and polyvinyl alcohol [CH_2_CH(OH)]_n_ 35 – 45% thickener purchased from BASF Germany. Sodium hexameta-phosphate, a dispersant, (Na_2_P_6_O_4,_ ≥ 68% P_2_O_5_) provided by Fine Chemicals.

### Preparation of Ag/ZnO modified nano pigments

Firstly, to prepare ZnO nanoparticles (solution A), 21.95 g of zinc acetate was dissolved in 250 ml of bi-distilled water in a beaker with agitation for 10 min. Ammonium hydroxide solution was added until the pH reached 9. Afterwards, the resulting solution was agitated for 30 min. Subsequently, a Büchner system was used to filter the prepared suspended particles, and they were washed various times with both distilled water and ethanol. The resulting ZnO pigment was then dried out at 70 °C for 12 h and calcined at 400 °C for 1 h. Secondly, Ag/ZnO nanopigments doped with 0.01% and 1% Ag nanoparticles were synthesized by adding, separately, the estimated quantity of AgNO_3_ to two distinct ZnO samples in a beaker containing 50 ml (0.01 mMol) of polyethylene glycol (PEG) and 50 ml (0.01 mmol) of polyvinylpyrrolidone (PVP). This mixture was then agitated for 10 min to prepare solution (B).

Solution (B) was mixed well with solution (A). After that, the mixture was heated to 80 °C for 2 h while being agitated in 10 ml of ethanol until the complete dissolution of silver salt. The prepared suspended particles were subjected to washing and centrifuging many times with distilled water, followed by ethanol. Subsequently, the precipitate was subjected to drying at 80 °C for 12 h.

### Preparation of Cu/ZnO modified nano pigments

In situ preparation of 0.01% and 1% Cu/ZnO nanoparticles was carried out as follows: 1 ml of ZnCl_2_ (0.1 mol) solution and 0.1 g sodium dodecyl sulfate were well blended with high agitation. Then, a calculated volume of 0.1 M CuCl_2_.2H_2_O solution was gradually mixed with ZnCl_2_ and a sodium dodecyl sulfate mixture and stirred for 3 h. Afterward, 1.8 ml of 1 M NaOH was added, followed by the addition of 8 ml of 0.1 M NH_2_OH. HCl, and the blend was agitated for 1 h. The formed precipitate was washed, centrifuged, and dried at 80 °C for 24 h. Finally, the formed precipitates were calcined at 400 °C for 1 h.

### Characterization

Both Ag/ZnO and Cu/ZnO nanopigments were examined employing various physiochemical techniques: The phases and crystallite sizes of Ag/ZnO and Cu/ZnO modified nanopigments were examined utilizing X-ray powder diffraction. The analysis was completed using a D/Max 2500 PC instrument from Rigaku, Japan (Cu-Kα radiation with a wavelength of 1.54059 Å and a target operating at 40 kV and 100 mA were utilized with a scan speed of 4°/min). The 2θ diffraction angle was adjusted in the range 10◦–80◦. An electron transmission microscope (TEM, Japan, JEM-2100) operating at 200 kV was used to analyze the particle morphology. The spectrofluorophotometer Shimadzu, RF-5301 PC (PL), Japan, and the Jasco-V-770 UV/VIS/NIR spectrophotometer, Japan, were used to examine the optical characteristics, luminescence spectrum and absorption spectra. Using Thermo Scientific K-Alpha X-ray photoelectron spectroscopy (XPS), the produced nanopigments’ valence state and elemental composition were determined. An Al-Ka micro-focused monochromator operating in the 4 keV energy range was employed with the XPS.

### Implementation of the synthesized nano pigments in paper coating

#### Paper coating mixtures preparation

The produced nanopigments were mixed for 20 min with a high shear mixer in distilled water containing 0.3 parts sodium hexametaphosphate as a dispersion agent, resulting in a 25% solid content. Subsequently, to prevent the creation of foam, the speed of the rotor was decreased to a moderate level while introducing 15 parts per hundred parts of binder, which was cautiously incorporated into the pigment slurry. Finally, NaOH solution was applied to raise the pH of the suspension to 8.

#### Coated paper samples preparation

**Fig. 1:**
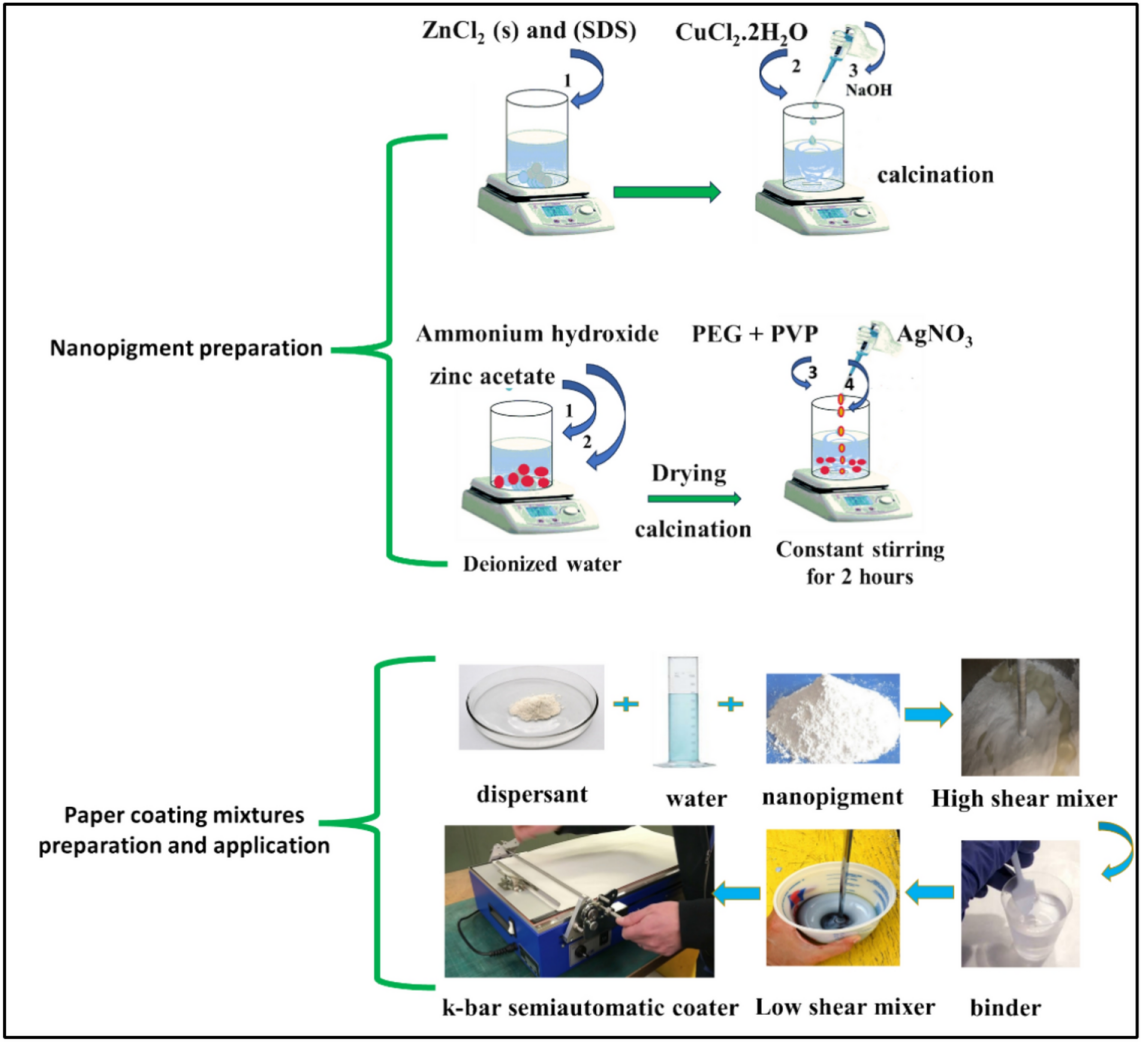
The complete process from nanopigment preparation to paper coating application.

The coating process was carried out using the k-bar, resulting in a wet layer thickness of 6 µm. Precoated paper having a grammage of 90 g/m^2^ and a dimension of 200 mm × 300 mm was used as a base paper. The samples were then coated in accordance with ISO 187 standard conditions, which are 23 ± 1 °C and 50 ± 2% humidity. Figure 1 illustrates the complete process from nanopigment preparation to paper coating application.

#### Characterization of coated paper

Coated paper is evaluated through various properties categorized into optical and mechanical aspects, each measured by specific standard methods and instruments. Brightness and Colorimeter device, model 68–59-00–002, manufactured by Buchel-B. V. in the Netherlands, was utilized to measure optical properties, brightness is measured as a percentage following the ISO 2470–1 (2009) standard. Whiteness is also assessed as a percentage according to ISO 11,476 (2010), while opacity is determined through ISO 2471 (2008). The tensile strength KN/mm, tensile energy absorption Jm^2^, and stretch percentage are quantified according to ISO 1924–2 (2008) with the T-series tensile test machine, model H5KT by Tinius Olsen Ltd., at 1KN. Burst strength, measured in Kpa is evaluated following ISO 2758–3 (2009) with a burst tester; model BT-10 by TlS Techlab Systems. Each of these measurements provides vital information on the quality and suitability of coated paper for various applications, ensuring that it meets the necessary standards and requirements for use in printing, packaging, and other industries.

#### Accelerated ageing

An accurate accelerated aging approach is preferred for assessing paper durability and anti-bacterial effect, as natural aging takes several years to reveal the significant changes in paper characteristics. The coated papers were heated for 29, 43, and 72 h at 100 °C in circulating air to simulate 10, 15, and 25 years of natural ageing. Before testing, the samples were conditioned according to TAPPI Method T 402.

#### Antimicrobial activity of accelerated ageing coated paper

The antimicrobial activity of previously aged paper samples was assessed using the Colony Forming Unit (CFU) assay. *Staphylococcus aureus* (*S. aureus*), known for causing skin- and food-related illnesses according to FDA guidelines, was selected as the pathogenic bacteria for testing^24^.

Mueller–Hinton broth medium was used for cultivating a suspension of *S. aureus* (McFarland standard 0.5) to assess the coated paper’s antibacterial efficiency. The aged paper samples and a DMSO control sample were then retained in a 96-well plate, and 200 μL of the resultant bacterial suspension was added. The CFUs were counted after an incubation duration of 24 h at 37 °C. The antimicrobial efficiency was calculated using the formula (N/N_o_) *100, where N_o_ and N represent the average CFU counts for the control substrate and aged paper sample, respectively. Colonies produced by the microorganisms on the plates were counted using a digital camera^25^.

#### Molecular docking of metal-doped nanoparticles with the S. aureus *MurA* enzyme

The crystal structure of the *S. aureus* MurA enzyme (UniProt ID: P84058) was obtained from the UniProt database and used for molecular docking studies. ZnO, Ag/ZnO, and Cu/ZnO nanoparticles were docked against the MurA enzyme using Molegro Virtual Docker, a molecular docking software. The docking simulations were accomplished to estimate the binding interactions and affinity of the nanoparticles with the MurA enzyme, a key enzyme involved in bacterial cell wall synthesis. The nanoparticles were designed using Materials Studio software.

#### Evaluation of metal-doped nanoparticles for inhibition of cell wall synthesis genes in *S. aureus*

The GeneMANIA tool was used to identify genes related to cell wall biosynthesis in *S. aureus*. The search was conducted using *S. aureus* as the organism and the key cell wall synthesis genes murF, mraY, murD, and murA as the input. The automatically selected weighting method was used to analyze the network of genetic interactions, co-expression, shared protein domains, and physical interactions.

#### Contact angle measurements

Water contact angles (WCA) are essential for assessing the effect of modified nanopigments on the hydrophobicity of paper surfaces. A hydrophobic surface is indicated by a water contact angle of greater than 90°, and a hydrophilic surface is suggested by an angle less than 90°. Complete wettability is characterized by a contact angle of 0°. Therefore, determining the contact angle is crucial and has practical implications in the food industry. The wettability of paper surfaces was measured using static contact angle assessments performed with the Angulus mobile application. After applying two μL droplets of liquid to the sample’s surface for five seconds at room temperature and controlled humidity, the WCA was determined using the sessile drop method.

#### Soil burial degradation test

A soil burial degradation test was carried out to assess the biodegradability of the coated paper. Small pieces of paper, measuring 20 × 20 mm, were buried in natural soil at a depth of 10 cm. After twelve days, the samples were collected, washed several times with distilled water, and then allowed to dry in an oven at 50 °C for 24 h. The weight losses of the samples were calculated after twelve days according to the following equation: Weight loss (%) = (M_0_ – M_i_) × 100, where M_i_ is the weight of the sample after twelve days degradation and M_0_ is the initial weight of the sample (g).

## Results and discussion

### Characteristics of the Ag/ZnO and Cu/ZnO modified nano pigments

**Fig. 2:**
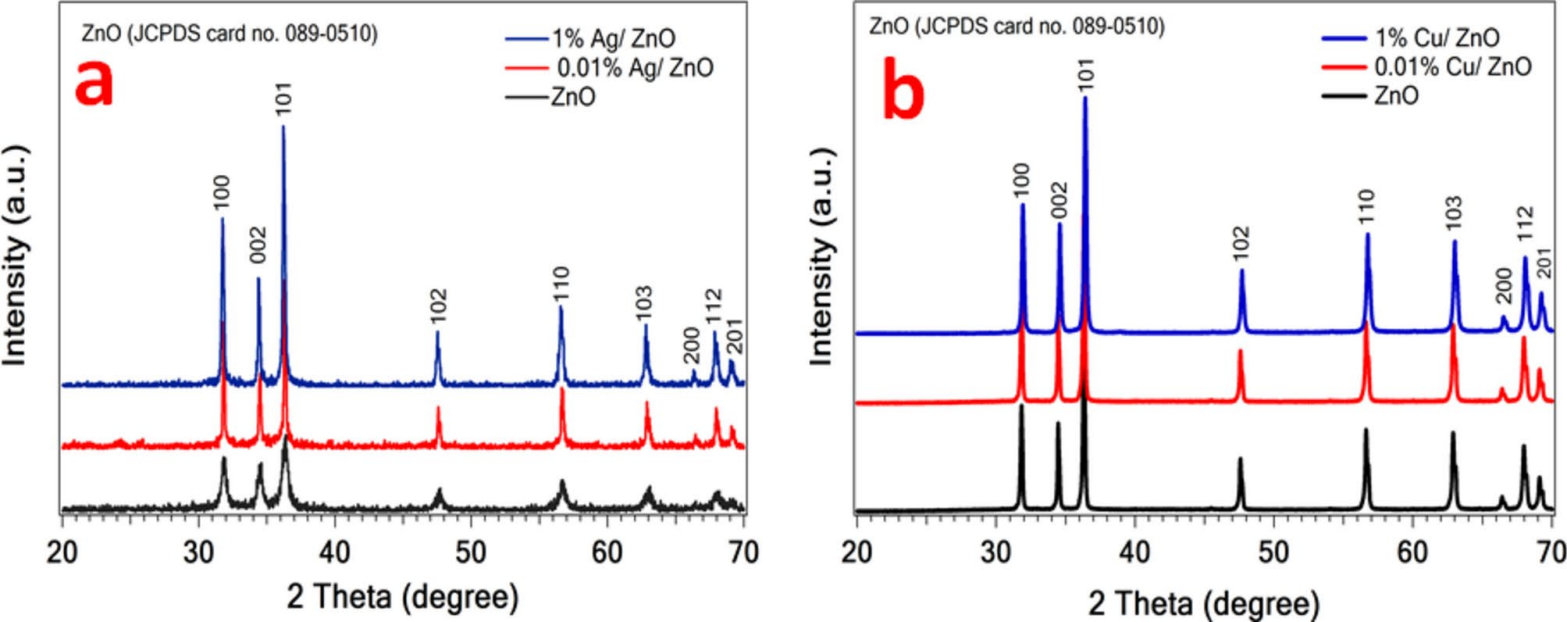
XRD patterns (**a**) Ag/ZnO and (**b**) Cu/ZnO samples.

Figure 2a, b illustrates the diffraction patterns of the prepared Ag and Cu/ZnO nanoparticles. It is clearly shown that all diffraction peaks of Ag/ZnO and Cu/ZnO samples are well matched and identified as ZnO phase (JCPDS card no. 089–0510). The 2-theta degree at 31.8, 34.5, 36.3, 47.6, 56.6, 62.9, 66.4, 68, 69.1 are consistent with the (100), (002), (101), (102), (110), (103), (200), (112), and (201) crystal planes orientation, respectively. No peaks are detected for Ag and Cu particles; this is associated with the very low Ag and Cu contents.

**Fig. 3:**
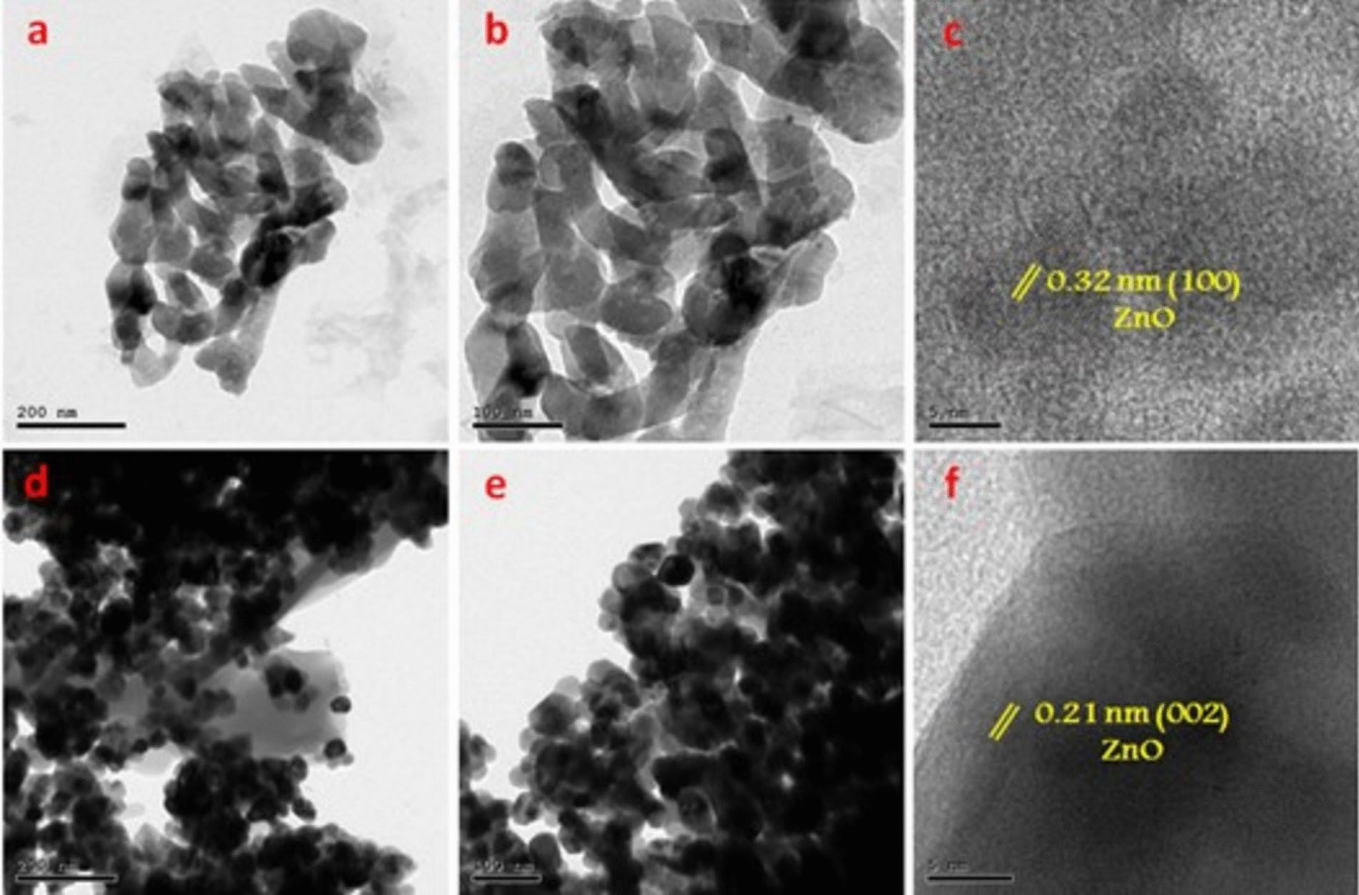
TEM and HRTEM images of (**a**,**b**,**c**) 1% Ag/ZnO and (**d**,**e**,**f**) 1% Cu/ZnO samples.

The TEM and HRTEM images of the as-prepared 1% Ag/ZnO and 1% Cu/ZnO nanoparticles are shown in Fig. 3 which shows the particle size is ranging from 20 to 50 nm. For the 1% Ag/ZnO sample, aggregated ZnO nanoparticles are clearly connected to each other, as seen in Fig. 3a, b. Obviously, the ZnO nanoparticles are decorated with small, spherical Ag nanoparticles. The lattice fringe of 0.32 nm is associated with the (100) crystal plane of Wurtzite ZnO, as seen in Fig. 3c ^26^. At the same time, TEM and HRTEM images confirmed the synthesis of the 1% Cu/ZnO nanoparticles with hexagonal structure of ZnO nanoparticles as shown in Fig. 3d, e; moreover, the lattice fringe of 0.21 nm is consistent with the (002) crystal plane of ZnO ^27^ as confirmed from the XRD data shown above.

**Fig. 4:**
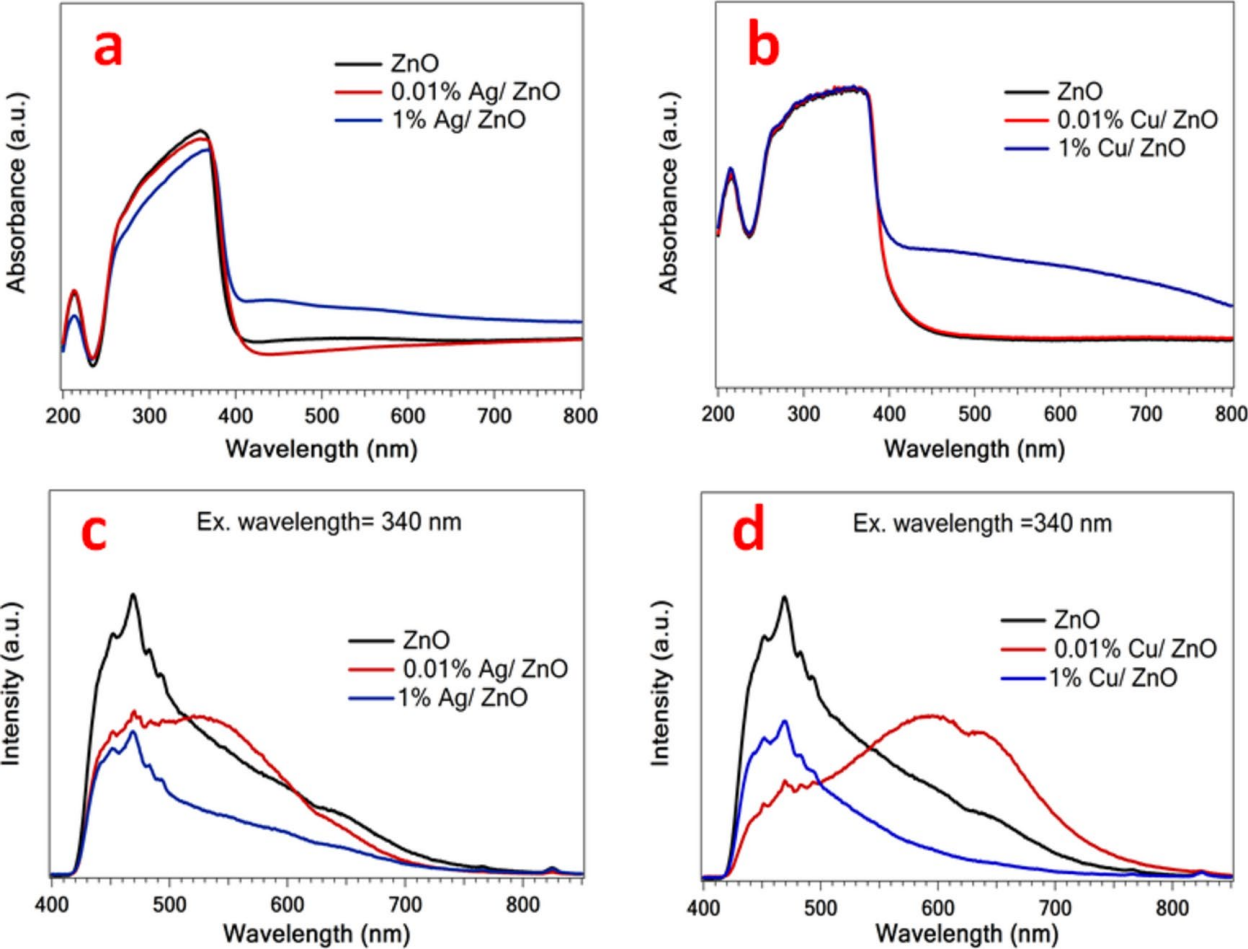
(**a**, **b**) UV–vis spectra and (**c**, **d**) PL spectra of ZnO, Ag/ZnO, and Cu/ZnO samples, respectively.

The optical properties of the Ag/ZnO and Cu/ZnO nanoparticles were investigated, as depicted in Fig. 4. Figure 4a, b display the distinctive absorption peak of ZnO at 360 nm for both samples. There is no big noticeable change in the plateau after the incorporation of Ag or Cu nanoparticles with ZnO; however, the absorption extends to the visible region. The PL spectra were measured at an excitation wavelength of 340 nm, as demonstrated in Figs. 4c, d. Clearly, the Ag/ZnO and Cu/ZnO samples exhibit the characteristic broad peak at around 470 nm. Briefly, the intensity peak tends to decrease with increasing the content of Ag or Cu particles in ZnO samples. The 1% Ag/ZnO and 1% Cu/ZnO samples performed the lowest intensity peaks compared to other samples, implying the best photocatalytic process, which is associated with excellent charge carrier separation. This would affect the antimicrobial activity as will be discussed later.

**Fig. 5:**
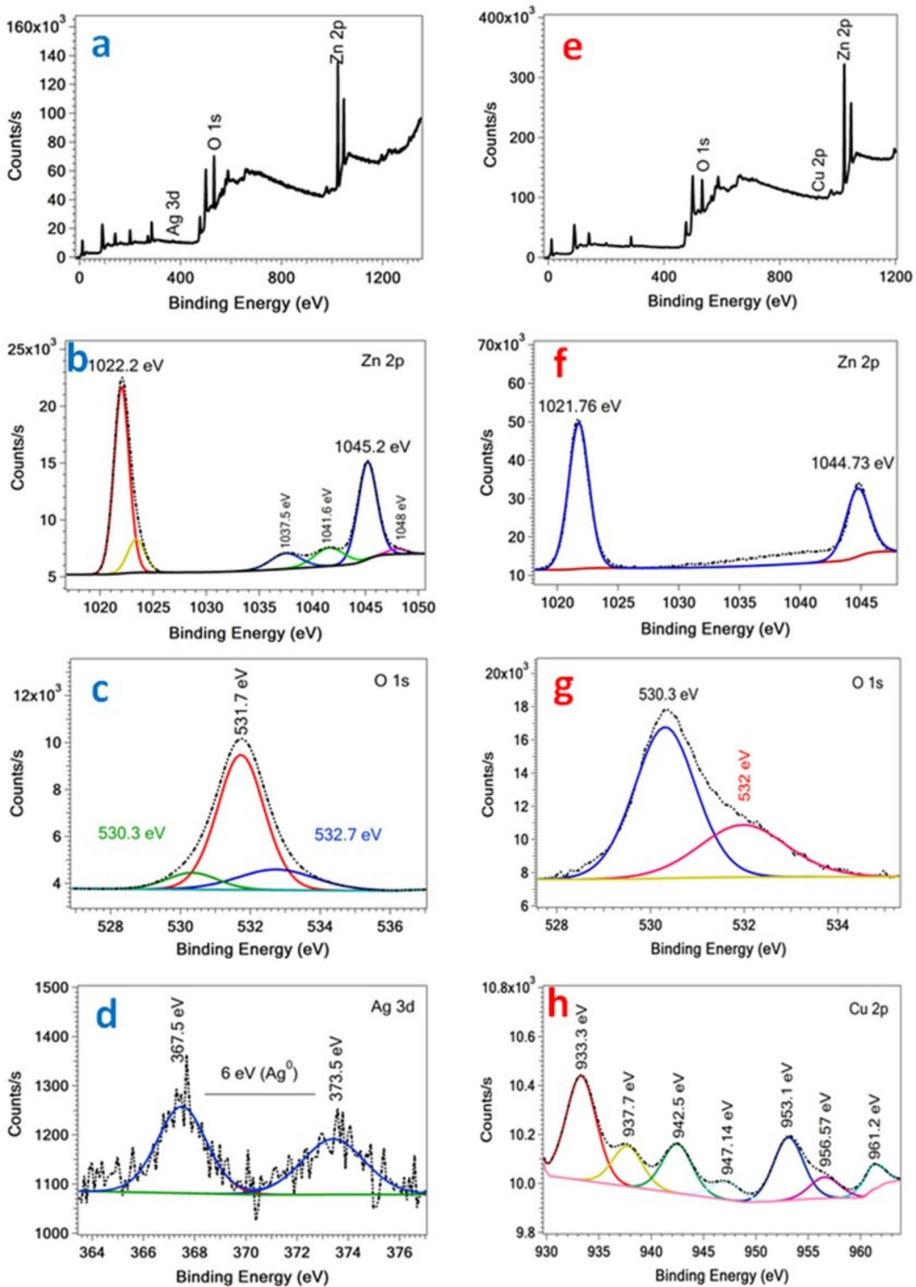
XPS spectra; (**a**) survey and high-resolution scan of (**b**) Zn 2p, (**c**) O 1 s, (**d**) Ag 3d of Ag/ZnO sample and (**e**) survey and high-resolution scan of (**f**) Zn 2p, (**g**) O 1 s, (**h**) Cu 2p of Cu/ZnO sample.

The chemical compositions and oxidation states of the as-prepared Ag/ZnO and Cu/ZnO samples were analyzed via XPS, as shown in Fig. 5. For Ag@ ZnO sample, the full scan confirmed the existence of Zn, O, and Ag elements (Fig. 5a). The Zn 2p splitting shows two binding energies at 1022.2 and 1045.2 eV, which are ascribed to Zn 2p3/2 and Zn 2p1/2, respectively (Fig. 5b). These values are the characteristic of Zn^2+^, which confirm the formation of ZnO phase^25^. The fitting of the O 1 s displays three peaks at 530.3, 531.7, and 532.7 eV as shown in Fig. 5c. The binding values at 530.3 and 531.7 eV are characteristics of Zn–O and O–H bonds, respectively^28,29^. The binding value at 532.7 eV is attributed to the existence of oxygen vacancies^30^. Figure 4d shows the splitting of Ag 3d into two peaks at binding energy values of 367.5 and 373.5 eV, which are ascribed to Ag 3d5/2 and Ag 3d3/2, respectively. Consequently, there is a 6 eV energy difference between the two peaks, which is characteristic of the oxidation state of Ag^0^^31^. On the other side, the full scan of the Cu/ZnO sample confirms the existence of Cu besides Zn and O elements, as shown in Fig. 5e. The two binding energy values at 1021.76 and 1044.73 eV are characteristics of Zn 2p3/2 and Zn 2p1/2, respectively^27^, confirming the oxidation state of Zn^2+^ as shown in Fig. 4f. The O 1 s XPS spectra (Fig. 5g) show two binding energy values at 530.3 and 532 eV. The peak at 530.3 eV could be assigned to Zn–O bond, while the peak at 532 eV is most likely related to the adsorbed oxygen ^28^. The Cu 2p splitting spectrum shows the characteristic peaks of Cu 2p1/2 and Cu 2p3/2 at 953.1 and 933.3 eV, respectively, as seen in Fig. 5h. These peaks indicate the trace amounts of the two oxidation states of Cu (I and II)^28,32^. Moreover, the peaks at binding energy values of 937.7, 942.5, 947.17, 956.57, and 961.2 eV are identified as satellite peaks.

### Application of the prepared pigments in paper coating

#### Optical properties

**Fig. 6:**
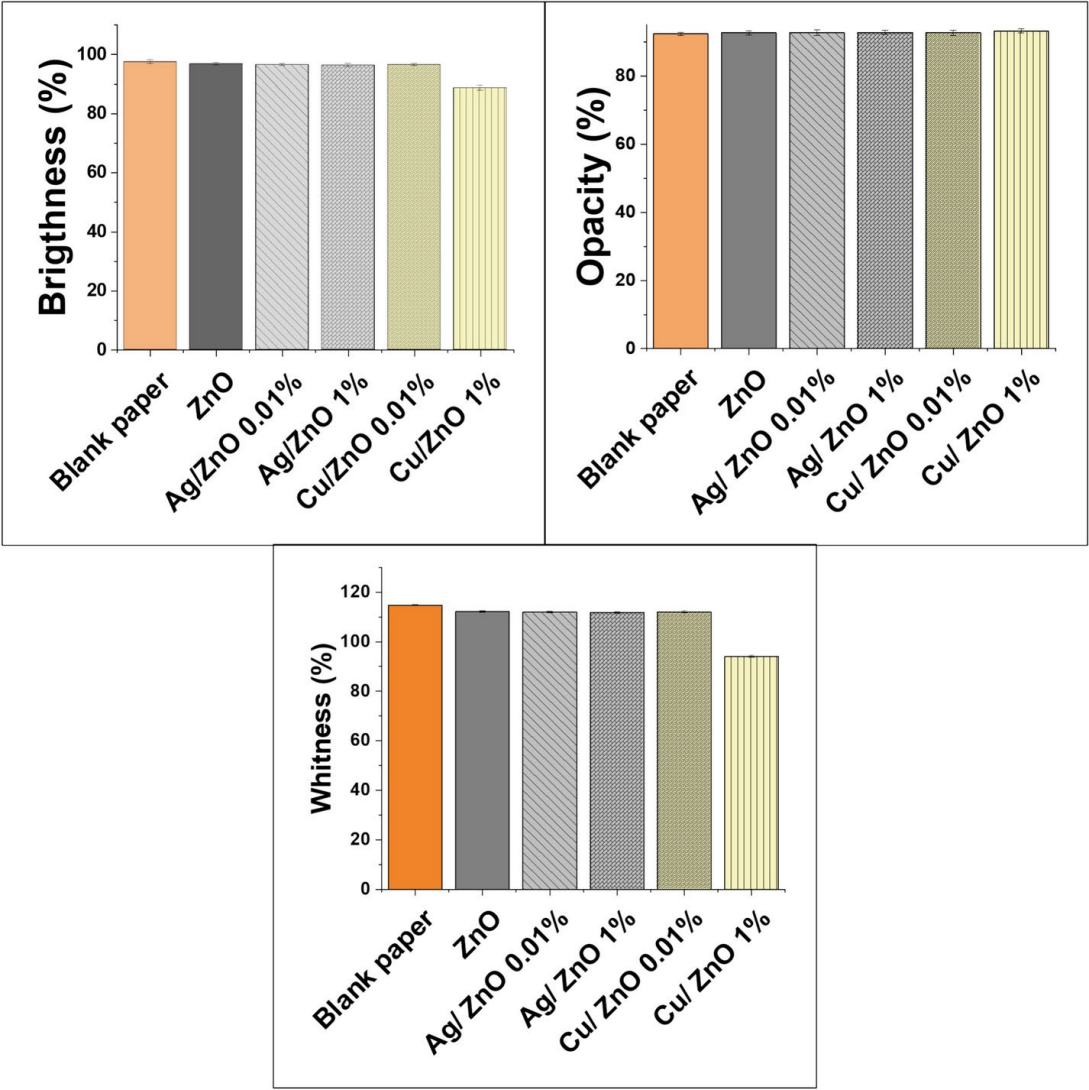
Optical properties of the prepared coated paper, including Brightness, Opacity, and Whiteness.

The optical properties of paper refer to how it interacts with light and how it appears visually. These properties are critical for various applications, including printing, packaging, publishing, and art. Some of the key optical characteristics of paper include brightness, color opacity, and whiteness^33–35^. The increasing demand for adding new functional characteristics to paper sheets, such as antimicrobial effect and strength, mustn’t negatively alter the paper’s optical quality, so it’s essential to consider the specific coating pigment and its compatibility with the coating mixture to achieve the desired results^4^. To deal with this issue, the impact of raising Ag content in Ag/ZnO and Cu content in Cu/ZnO-modified nanopigments on paper optical quality was examined. The prepared coated paper’s optical characteristics are displayed in Fig. 6. Despite the gray black colour of the Ag nanoparticles, the results showed that raising the Ag content to 1% in Ag/ZnO had no significant influence on the optical characteristics of the coated paper when compared with pure ZnO.

The little amount of Ag-doped coated paper and how well it is spread out in the ZnO matrix porous system may explain these better qualities. Increasing the content of Cu to 1% in Cu/ZnO has no substantial impact on the paper’s opacity while decreases brightness and whiteness by 8.5% and 16%, respectively. These results are attributed to the relatively small energy band gap of Cu ^36,37^ that tends to absorb longer wavelengths (lower frequencies) of light, which often results in a darker color appearance.

#### Mechanical properties

**Fig. 7:**
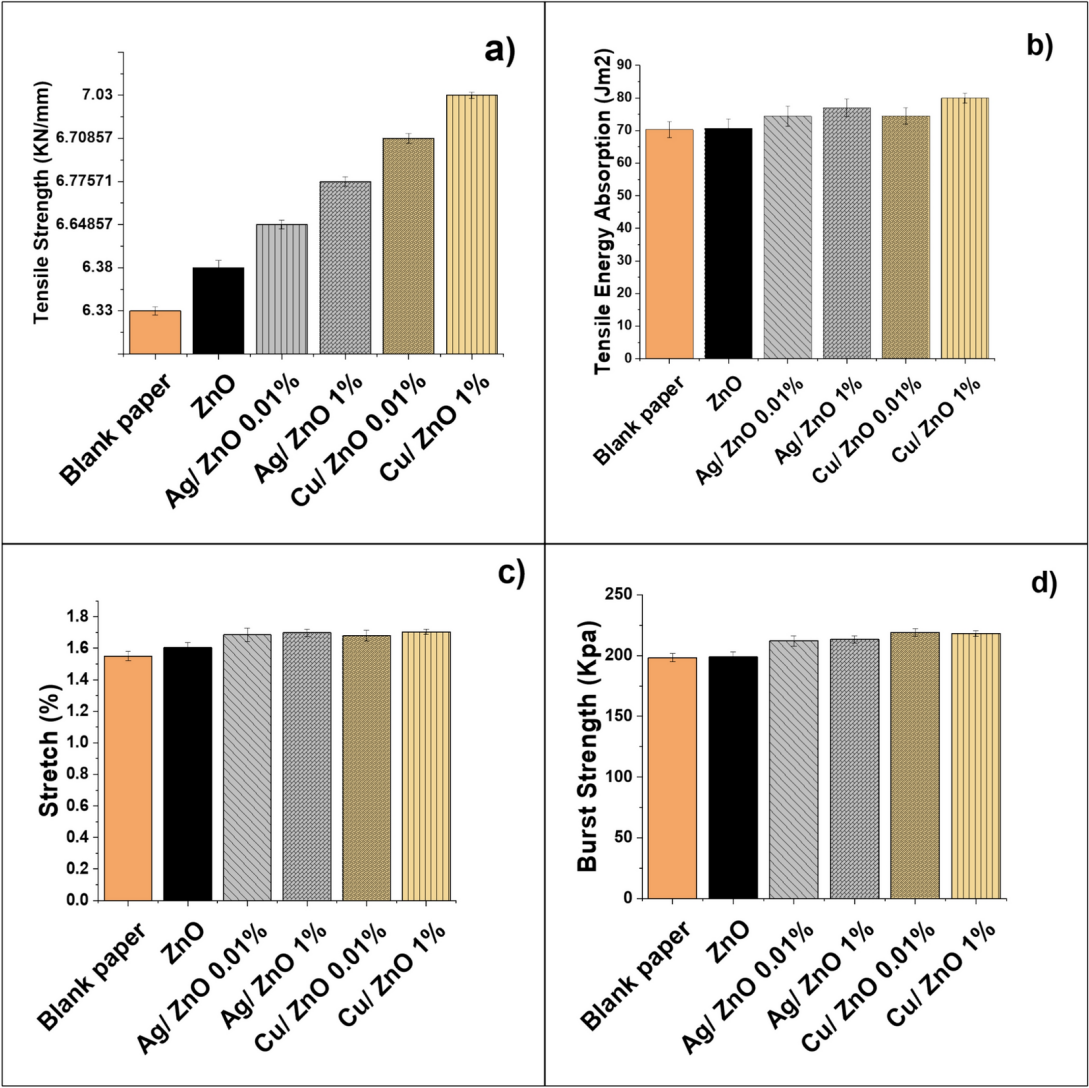
The prepared coated paper’s mechanical properties (**a**) tensile strength (**b**) stretch (**c**) tensile energy absorption (**d**) burst strength.

In the paper industry, it is well known that the type and formulation of the coating mixture as well as the base paper have a great impact on the mechanical structure of the final coated paper. These mechanical properties refer to the characteristics that describe how paper withstands applied forces or loads. Various coating mixtures may be combined for different applications, such as high-quality printing, magazine production, packaging, or hygiene paper, to ensure optimal performance and functionality^38^. Figure 7 and Table 1 illustrate the mechanical properties of the prepared coated paper. The tensile strength and stretch of ZnO-coated paper are 6.34 kN/m and 1.60%, respectively. The tensile strength enhanced significantly with higher Ag and Cu content in the Ag/ZnO and Cu/ZnO coatings compared to the ZnO-coated paper. As illustrated in Fig. 7a, with Ag and Cu content raised to 1%, the tensile strength reached a maximum of 6.77 kN/m and 7.03 kN/m, representing improvements of approximately 6.7% and 10.8%, respectively. Concurrently, the stretch enhanced to 1.69% and 1.70%, reflecting improvements of about 5.6% and 6.25%, as appeared in Fig. 7b. Tensile energy absorption (TEA) is quantified by the ability of paper to absorb energy under different types of force, which helps in selecting the appropriate paper grades and understanding how they will perform under tensile stress and the potential for tearing or failure^39^. Figure 7c illustrates that TEA of the ZnO-coated paper increased from 70.60 J/m^2^ to about 77 and 80 J/m^2^, with an improvement of nearly 10 and 13.4% using 1% Ag/ZnO and Cu/ZnO nanopigments, respectively. The burst strengths of the coated papers exhibited a similar trend, increasing with higher Ag and Cu nanoparticle content. The burst strength of paper coated with 1% Ag@TiO_2_ and Cu/ZnO modified nano pigment increased by a percentage of 9.7 and 10.2% compared with pure ZnO coated paper, as illustrated in Fig. 7d, which allows the paper to withstand greater pressure without bursting or delaminating. It should be mentioned that Cu/ZnO-coated paper has higher mechanical properties than Ag/ZnO coated paper. These results are attributed to the fact that the pigment crystal structure plays a significant role in determining a coating’s mechanical property^4^. The crystal structure of Cu contributes to its higher hardness compared to Ag, as the measured hardness data of pure Ag and Cu metal are 2.5 and 3 on the mohs scale, respectively. The monoclinic crystal structure of Cu leads to fewer slip planes for atomic movement and a strong, rigid crystal structure compared to the face-centered cubic (FCC) structure of Ag.

**Table 1:** Mechanical properties of the prepared coated paper.

Mechanical properties	Blank paper	ZnO coated paper	0.01% Ag/ZnO	1% Ag/ZnO	0.01% Cu/ZnO	1% Cu/ZnO
Tensile strength (KN\mm)	6.33 ± 0.1	6.34 ± 0.16	6.64 ± 0.10	6.77 ± 0.10	6.71 ± 0.18	7.03 ± 0.06
Stretch%	1.55 ± 0.03	1.60 ± 0.03	1.68 ± 0.04	1.69 ± 0.02	1.68 ± 0.03	1.70 ± 0.01
Tensile energy absorption (J/m^2^)	70.3 ± 2.5	70.60 ± 2.84	74.38 ± 3.03	77 ± 4.37	74.48 ± 2.15	80 ± 1.48
Burst strength (Kpa)	198.3 ± 3.5	198.79 ± 4.40	212.28 ± 4.22	218.15 ± 3.01	213.35 ± 3.29	219 ± 2.41

#### Antimicrobial activity tests for the aged coated paper samples

**Fig. 8:**
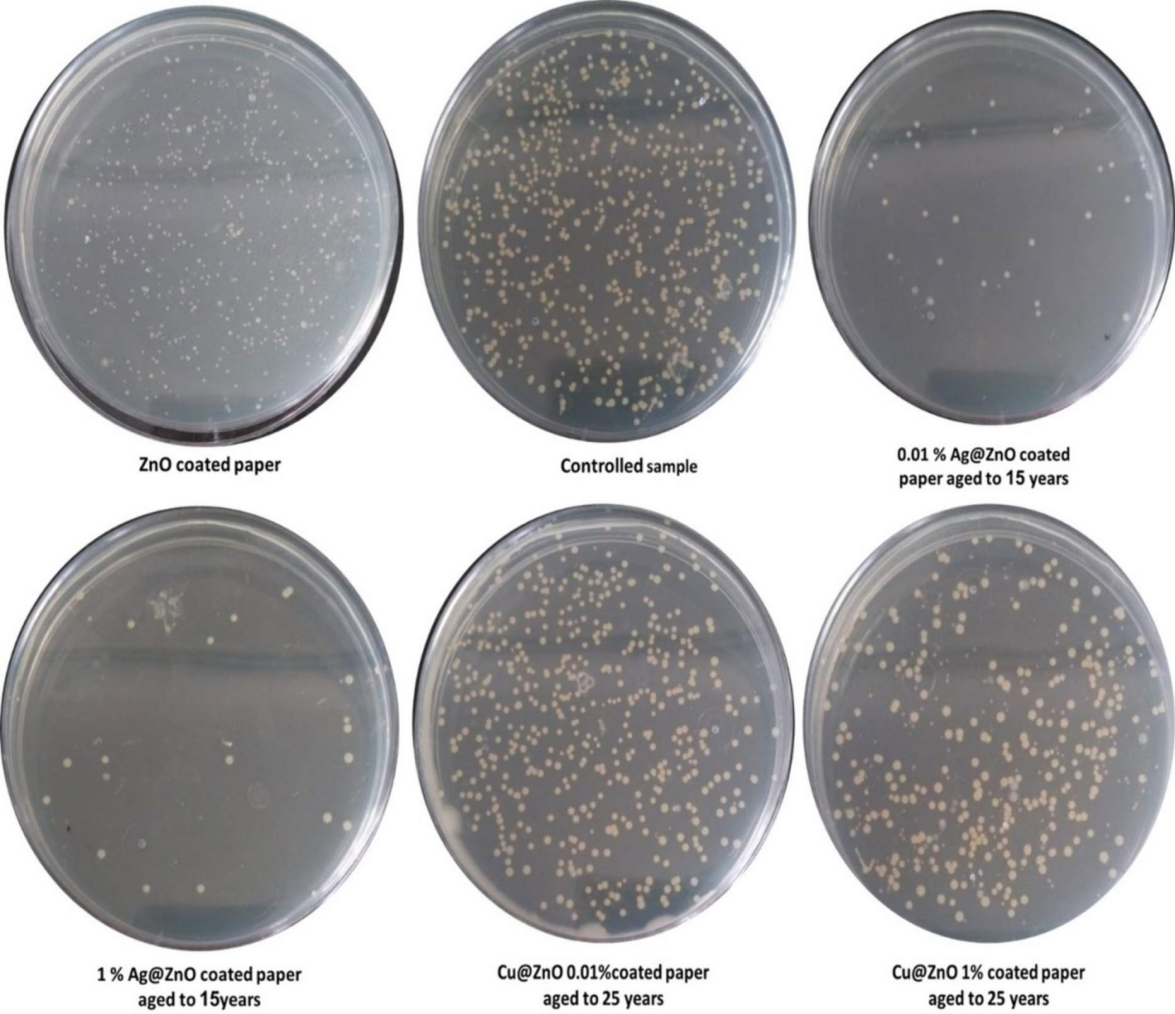
Antimicrobial activity of aged, coated paper samples containing 0.01% and 1% Ag/ZnO and 0.01% and 1% Cu/ZnO nanoparticles.

**Fig. 9:**
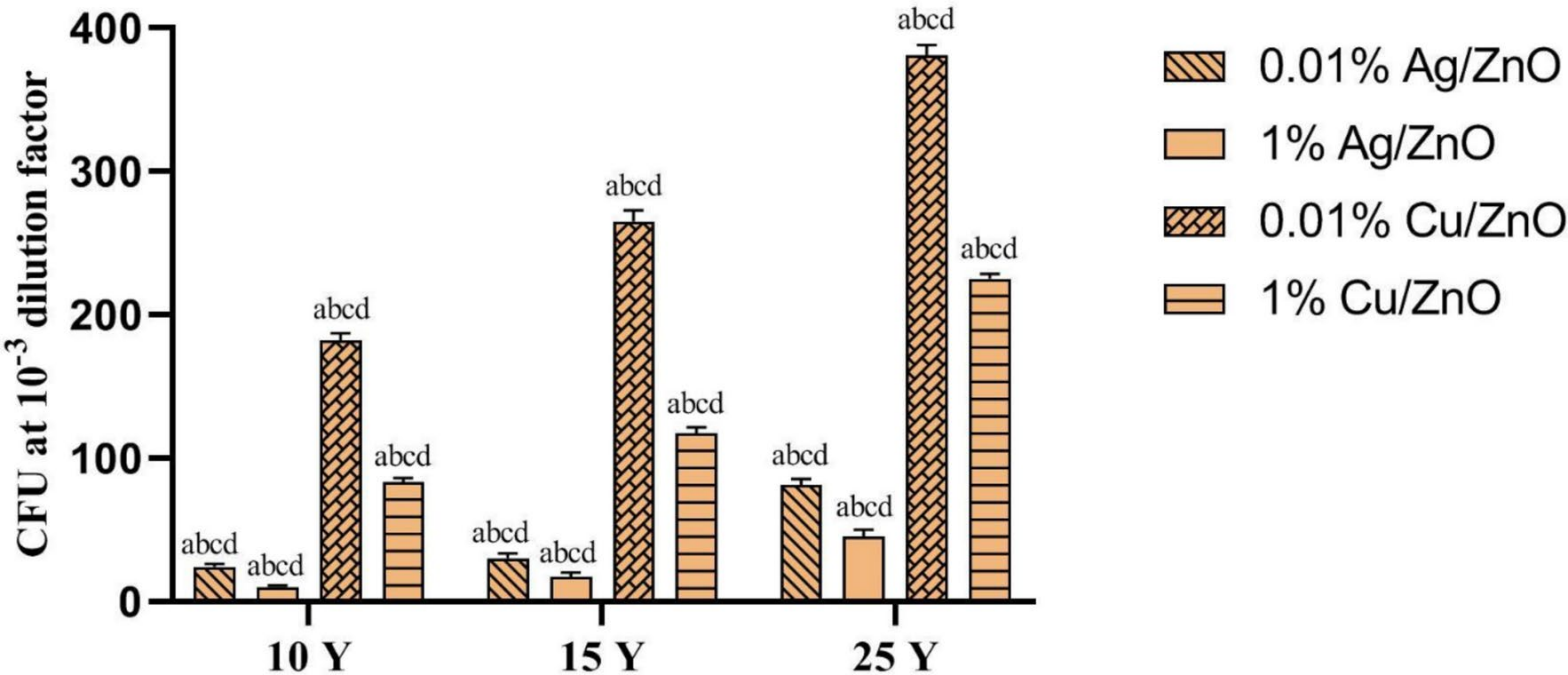
Colony forming unit of coated paper samples containing 0.01% and 1% Ag/ZnO and Cu/ZnO nanoparticles at 10, 15, and 25Y. Statistical analysis difference between obtained values (mean ± SD) were carried out by Two-Way analysis of variance (Anova) followed by Bonferroni’s multiple comparison test. A value of 0.05 or less was taken as criterion for a statistical difference. Graphpad prism 8was used for statistical analysis. a is significant difference from 0.01% Ag/ZnO, b is significant difference from 1% Ag/ZnO, c is significant difference from 0.01% Cu/ZnO and d is significant difference from 1% Cu/ZnO.

The colony-forming units CFU assay test results show that the addition of Ag and Cu nanoparticles to the Ag/ZnO and Cu/ZnO coatings significantly enhanced the inhibition effect and decreased the colony-forming units. The paper coated with 1% Ag/ZnO showed an inhibition percent of 92.2%, while the paper coated with 1% Cu/ZnO reached 62.2% (Table 2, Fig. 8). The use of Ag and Cu nanoparticles in the ZnO surface coating of coated paper has been shown to enhance its antimicrobial properties, particularly in industries where hygiene is crucial^40^. Figure 9 demonstrated that CFU decrease with raising Ag content in Ag/ZnO and Cu content in Cu/ZnO-modified nanopigments. Although both silver (Ag) nanoparticles and copper nanoparticles have demonstrated antimicrobial properties, samples doped with Ag nanoparticles remain active against bacteria better than samples coated with Cu^41^. The variance in antimicrobial behavior is attributed to the differing amounts of released ions, silver nanoparticles release a higher amount of silver ions, which are thought to be the primary antimicrobial agent and can disrupt bacterial cell membranes, interfere with cellular processes, and generate reactive oxygen species that damage bacterial cells, whereas copper nanoparticles release a lower amount of copper ions that have antimicrobial properties but not to the same degree, likely explaining why samples doped with silver nanoparticles demonstrate more sustained antimicrobial activity, as the higher silver ion concentrations allow the silver nanoparticle-containing samples to maintain their antibacterial efficacy for longer^42^. The two-way ANOVA test revealed a statistically significant difference in the antibacterial activity of the various ZnO-based nanoparticles against *S. aureus* (p-value ˂0.05). The CFU values, representing bacterial survival, varied significantly between the treatment groups, indicating that doping nanoparticles had distinct effects on bacterial inhibition. Pulit-Prociak et al. (2020)^43^ found that zinc oxide, silver, and copper nanoparticles exhibited antibacterial properties against the Gram-positive bacterium *S. aureus*. The biocidal effect was conferred by the incorporation of the metal oxide and metallic nanoparticles.

**Table 2:** Antimicrobial activity of aged, coated paper samples containing 0.01% and 1% Ag/ZnO and 0.01% and 1% Cu/ZnO nanoparticles.

*Staphylococcus aureus*	ZnO coated paper	0.01% Ag@ZnO			1% Ag@ZnO			0.01% Cu@ZnO			1% Cu@ZnO			Control (DMSO)
		10 Y	15 Y	25 Y	10 Y	15 Y	25 Y	10 Y	15 Y	25 Y	10 Y	15 Y	25 Y	
Dilution Factor	10^−3^	10^−3^	10^−3^	10^−3^	10^−3^	10^−3^	10^−3^	10^−3^	10^−3^	10^−3^	10^−3^	10^−3^	10^3^	10^−3^
Volume of broth plated (µl)	20	20	20	20	20	20	20	20	20	20	20	20	20	20
(CFU) at the dilution factor	404 ± 17.35	24 ± 2.00	30 ± 3.61	81 ± 4.58	10 ± 1.00	17 ± 3.00	45 ± 5.20	182 ± 5.29	265 ± 7.94	381 ± 7.00	83 ± 3.00	117 ± 4.35	225 ± 3.60	580 ± 5.56
Total CFU /ml	15,150,000 ± 650,600.68	900,000 ± 75,000.00	1,125,000 ± 135,208.17	3,037,500 ± 171,846.59	375,000 ± 37,500.00	637,500 ± 112,500.00	1,687,500 ± 135,208.17	6,552,000 ± 198,431.34	9,937,500 ± 297,647.02	14,287,500 ± 262,500.00	3,112,500 ± 112,500.00	4,387,500 ± 163,458.71	8,437,500 ± 135,208.17	21,750,000 ± 208,791.16
Log total CFU	7.18	5.95	6.05	6.48	5.57	5.81	6.23	6.82	7.00	7.16	6.49	6.64	6.93	7.34
Percent of Inhibition%	30.4	95.9	94.8	86.04	98.3	97.1	92.2	69.9	54.3	34.3	85.7	79.8	61.2

#### Possible mechanism for antibacterial activity

The antibacterial action of Ag and Cu-doped ZnO nanoparticles can be attributed to a combination of physical and chemical interactions with bacterial cells, primarily involving the generation of reactive oxygen species (ROS), release of metal ions, and direct interaction with the cell membrane and DNA. These mechanisms are summarized below.

## Production of reactive oxygen species (ROS)

Ag@ZnO and Cu@ZnO nanoparticles generate ROS, such as hydroxyl radicals (^*^OH), superoxide anions (O_2_^•−^), and hydrogen peroxide (H_2_O_2_), which induce oxidative stress in bacterial cells. ZnO nanoparticles, when irradiated with UV or visible light, excite electrons from the valence band to the conduction band, leaving behind a hole (h^+^) that can participate in the formation of ROS ^44^.

The following reactions can describe the ROS generation mechanism:

### a. Excitation of ZnO under light:

ZnO (Ag, Cu doped) + *hv*(light) → e^−^ (conduction band) + *h*^+^ (valence band).

### b. Formation of hydroxyl radicals (^*^OH):

*h*^+^  + H_2_O → ^*^OH + H^+^

### c. Formation of superoxide anions (O_2_^•−^):

O_2_ + e^−^  → O_2_^•−^

### d. Formation of hydrogen peroxide (H_2_O_2_):

O_2_^•−^  + 2H^+^ → H_2_O_2_.

These ROS species cause oxidative damage to bacterial lipids, proteins, and DNA, disrupting normal cellular functions and leading to bacterial cell death^45^.

## Disruption of membrane Integrity

Ag^+^ and Cu^2+^ ions, released from the doped ZnO nanoparticles, bind to bacterial membranes and disrupt their integrity. The ions interact with membrane proteins and lipids, causing cellular contents to leak, ultimately leading to cell lysis.

Ag^+^  + Membrane proteins → Protein denaturation and cell lysis.

The positively charged ions interact with negatively charged bacterial membranes, altering their permeability and causing irreversible damage^46^.

## Interaction with bacterial DNA

Once inside the bacterial cell, Ag^+^ ions bind to bacterial DNA and interfere with its replication. This interaction prevents the bacteria from reproducing and can lead to mutations that further impair cell function.

Ag^+^  + DNA → DNA denaturation and inhibition of replication.

## Synergistic antibacterial effects of Ag and Cu

**Fig. 10:**
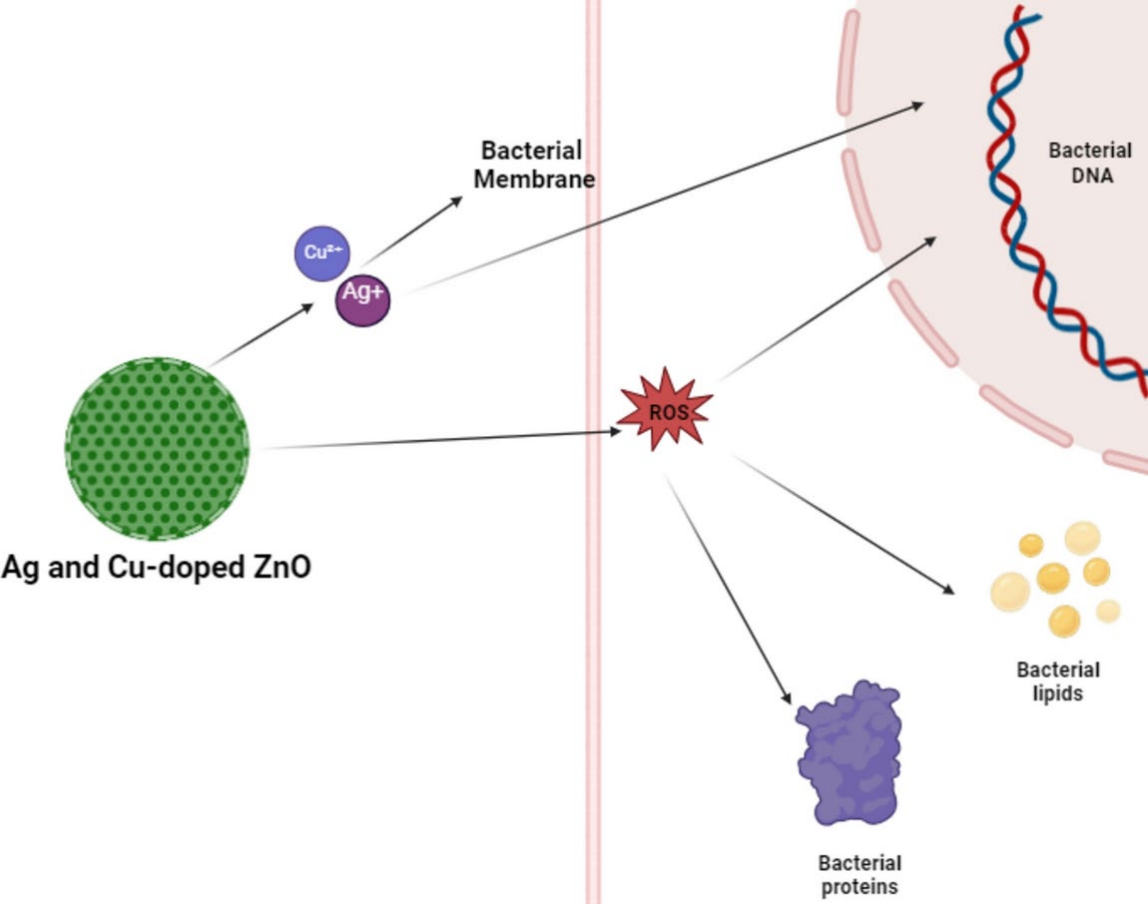
Graphical representation of antibacterial mechanisms of Ag and Cu-doped ZnO nanoparticles, including ROS generation, membrane disruption, and DNA interaction.

As shown in Fig. 10, the combination of Ag and Cu in ZnO nanoparticles enhances antibacterial activity by targeting different aspects of bacterial physiology. Ag generates ROS and binds to membrane proteins, while Cu can catalyze further ROS formation and directly interfere with bacterial enzymes. This synergistic effect leads to a higher overall antibacterial efficacy^47^.

## Molecular docking of metal-doped nanoparticles with the S. aureus *MurA* enzyme

**Fig.11:**
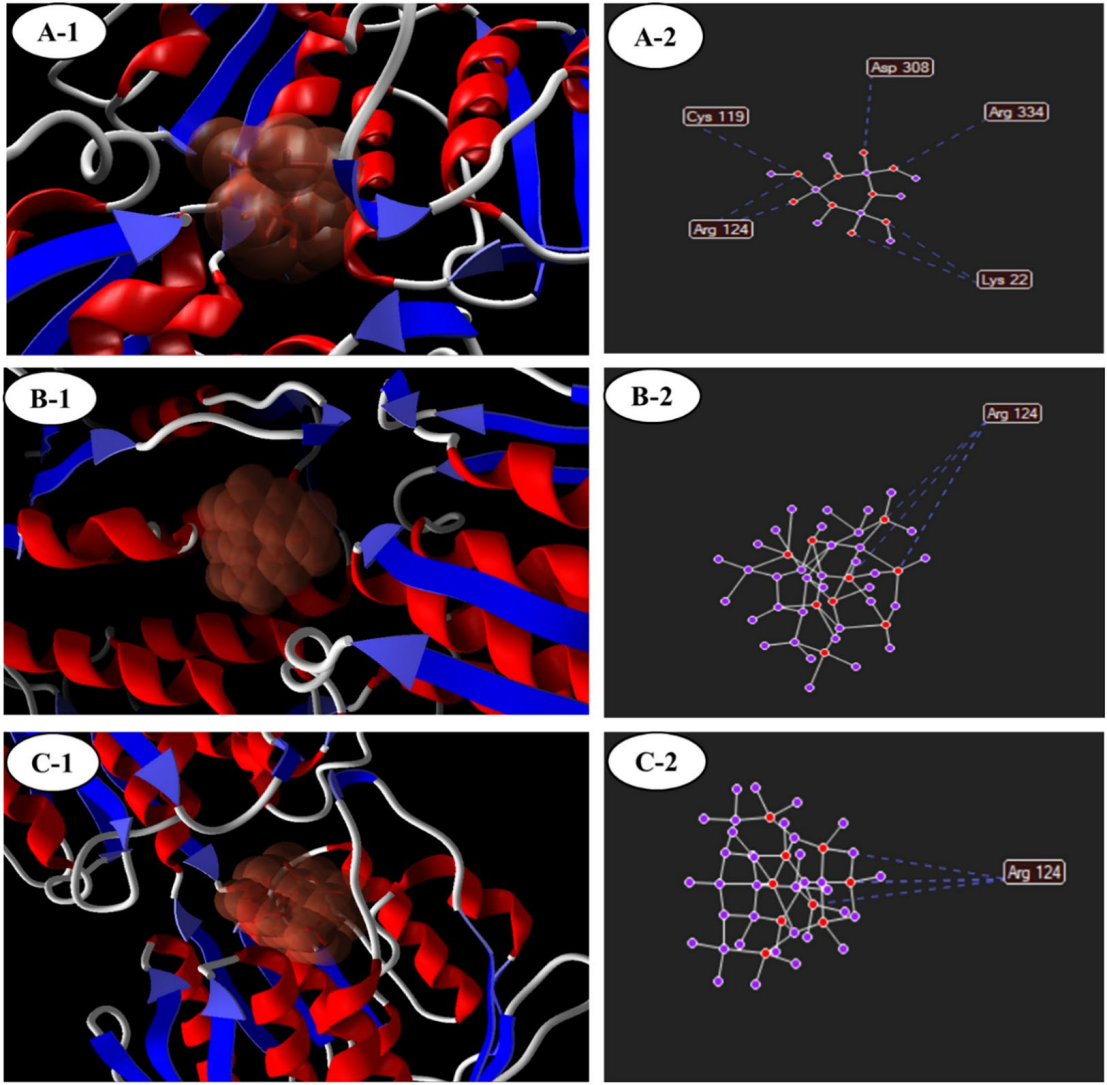
Molecular docking of nanoparticles with the Staphylococcus aureus MurA enzyme (**A**) ZnO nanoparticles, (**B**) Ag/ZnO nanoparticles, and (**C**) Cu/ZnO nanoparticles.

The molecular docking studies revealed that the ZnO nanoparticle formed extensive hydrogen bond interactions with several crucial residues of the *S. aureus* MurA enzyme, including Lys 22, Cys 119, Arg 124, Arg 308, and Arg 334, and exhibited a docking score of -41.276 kcal/mol, suggesting that the ZnO nanoparticle has the potential to disrupt the enzymatic function of MurA, which catalyzes the first committed step in peptidoglycan biosynthesis, a critical component of the bacterial cell wall. By interfering with this essential cell wall synthesis pathway, the ZnO nanoparticles may exhibit potent antibacterial activity against *S. aureus*, including drug-resistant strains. The Ag/ZnO nanoparticles showed a docking score of -21.835 kcal/mol and interacted with the Arg 124 residue of the MurA enzyme, indicating a possible inhibitory effect on cell wall synthesis, while the Cu/ZnO nanoparticles had a docking score of -21.842 kcal/mol and similarly interacted with the Arg 124 residue, suggesting a potential role in disrupting the MurA enzyme activity and, consequently, the bacterial cell wall biosynthesis. Inhibition of the MurA enzyme can be an effective strategy to compromise the bacterial cell wall integrity and viability, and the insights from this molecular docking study provide a foundation for understanding the possible mechanisms of action of these metal-doped nanoparticles against bacterial cell wall biosynthesis (Fig. 11). Alam (2021)^48^ investigated the antimicrobial properties of biogenic zinc oxide nanoparticles (ZnO nanoparticles) against the clinically important pathogen *S. aureus*. The study found the biogenic ZnO nanoparticles demonstrated significant antimicrobial activity against S. aureus, attributed to the nanoparticles’ ability to interact with and inhibit the bacterial cell wall, as confirmed through molecular docking analysis.

## Evaluation of metal-doped nanoparticles for inhibition of cell wall synthesis genes in *S. aureus*

**Fig. 12:**
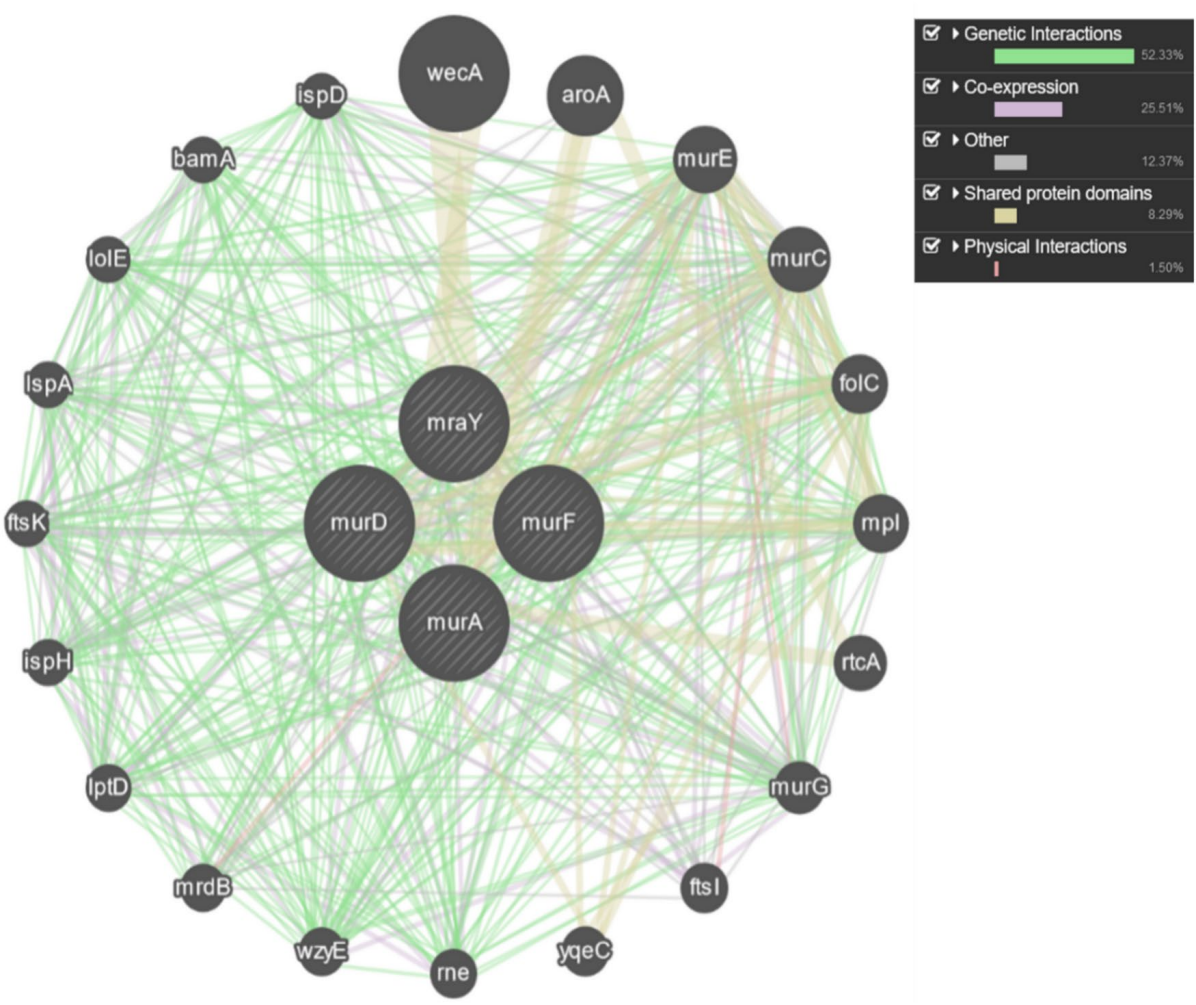
Network of genes functionally associated with cell wall synthesis in *S. aureus.*

The GeneMANIA analysis revealed a comprehensive network of genes that are functionally associated with the key enzymes involved in peptidoglycan biosynthesis in *S. aureus*, including murF, mraY, murD, and murA. These genes play critical roles in various stages of cell wall synthesis, underscoring their essential contribution to bacterial cell envelope integrity and viability. The strong genetic interactions, co-expression patterns, and physical interactions observed between these core cell wall synthesis genes and the broader network of associated genes highlight the intricate regulatory mechanisms and functional interdependence within the bacterial cell envelope biogenesis pathways. The identified genes encompass a diverse array of cellular processes, such as cell division, outer membrane biogenesis, and stress response, emphasizing the central importance of maintaining cell wall integrity for bacterial survival and proliferation. In this context, the evaluation of ZnO, Ag/ZnO, and Cu/ZnO nanoparticles as potential antimicrobial agents is warranted, as these nanoparticles may exert their inhibitory effects through direct interference with cell wall biosynthesis enzymes, disruption of the coordination between cell wall synthesis genes and other essential pathways, or compromising the overall structural and chemical integrity of the bacterial cell envelope, leading to cell lysis and death (Fig. 12). Guan et al. (2021)^49^ found that the ZnO nanoparticles inhibited *S. aureus* bacterial growth. Transcriptomic analysis revealed that the differentially expressed genes were involved in cell wall and membrane synthesis, indicating the film’s ability to disrupt the cell wall integrity of *S. aureus*. The changes in gene expression contributed to the high inhibition of *S. aureus* by the ZnO nanoparticles.

### Water contact angle

**Fig. 13:**
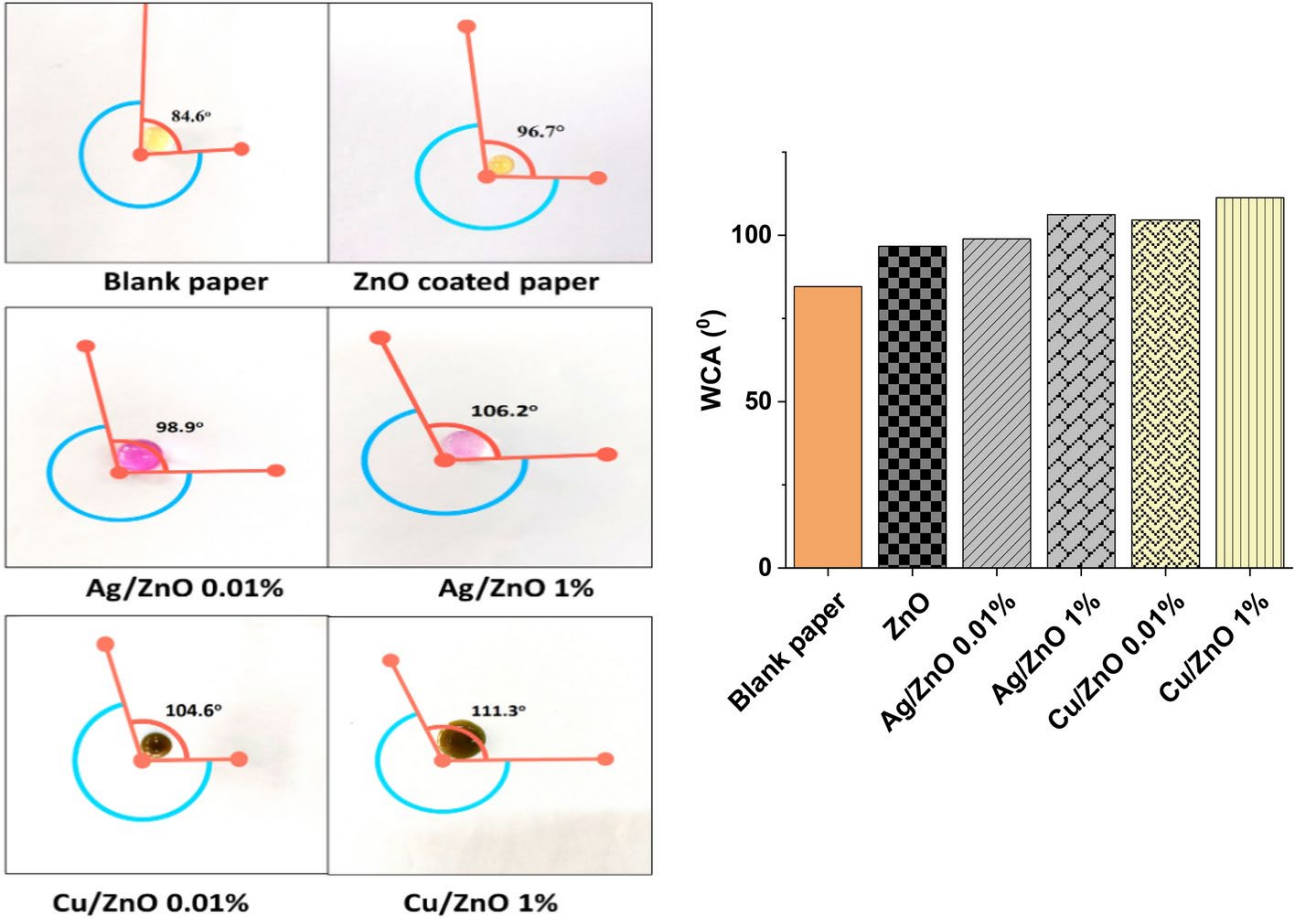
Water contact angle measurements of coated paper samples with 1% and 0.01% Ag/ZnO and Cu/ZnO nanoparticles.

Figure 13 illustrates the water contact angle of cellulosic paper that has been coated with modified nano pigments. The uncoated, blank paper demonstrates a lower water contact angle of 84.6°. This is attributed to the cellulosic composition and porous microstructure of the paper, which naturally tend to absorb water, thus reducing the contact angle^50^. Coating paper with ZnO nanopigments results in an increased water contact angle of 96.7°. The water contact angle values for paper coated with Cu/ZnO are higher in comparison to those coated with Ag/ZnO at the same concentration. Cu/ZnO creates a surface with lower energy, making it more hydrophobic, hence increasing the water contact angle. This could be due to the chemical activity nature of Cu, which, when combined with ZnO, might promote more hydrophobic interactions compared to Ag/ZnO^51–53^.

### Biodegradability of the coated papers

**Fig. 14:**
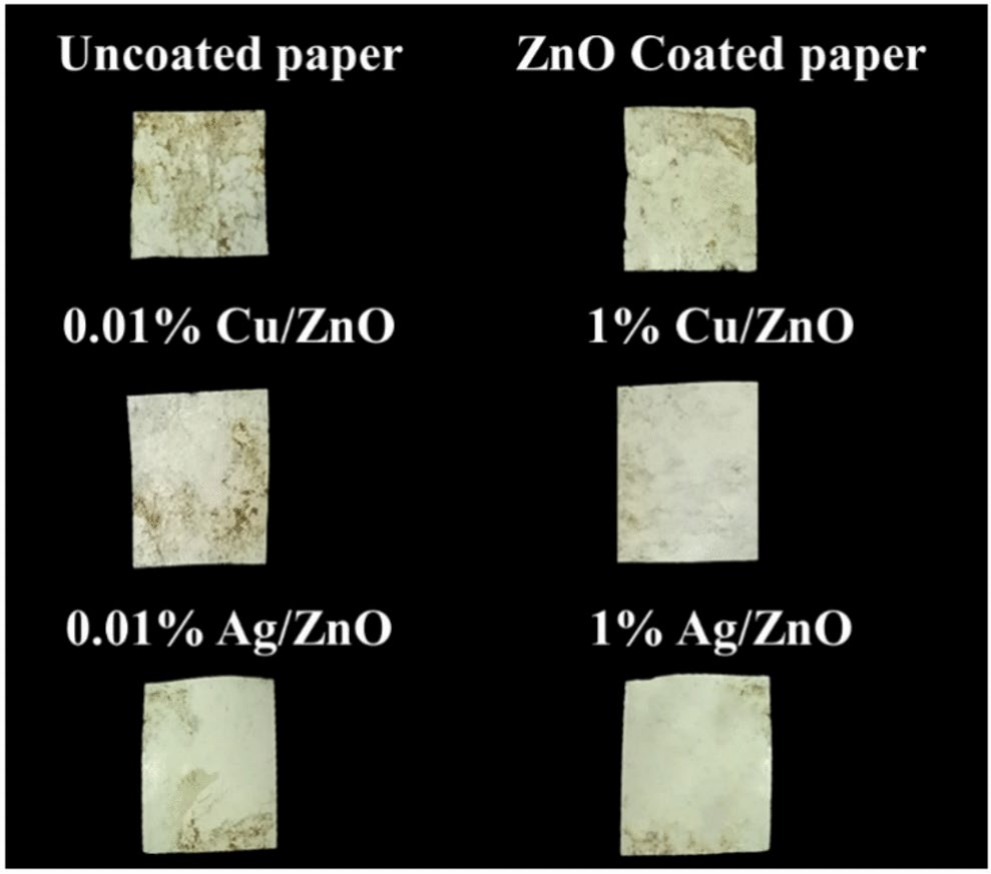
Biodegradation of coated paper samples after twelve days of soil burial test.

As shown in Fig. 14 and Table 3, uncoated paper exhibited the highest weight loss (0.30%), while ZnO-coated paper showed a moderate weight loss (0.23%). Papers coated with 0.01% and 1% Cu/ZnO also experienced considerable degradation, with weight losses of 0.21% and 0.15%, respectively. In contrast, silver-coated samples showed much lower biodegradation, with 0.01% Ag/ZnO losing only 0.08% of its weight and 1% Ag/ZnO exhibiting the least weight loss at 0.03%. These findings demonstrate that different coatings impact the biodegradability of paper, with uncoated paper degrading the most and Ag/ZnO-coated samples degrading the least. Notably, the higher concentration of silver (1% Ag/ZnO) significantly reduced biodegradability due to silver’s antimicrobial properties^54^. The samples would likely experienced biodegradation at varying rates depending on the coating^55^.

**Table 3:** Weight Loss (%) of paper samples with different coatings after twelve days of soil burial test.

Sample	Weight (g)		Weight Loss (%)
	Initial weight	Final weight	
Uncoated paper	0.0382 ± 0.0002	0.0352 ± 0.0003	0.30
ZnO	0.0393 ± 0.0003	0.0370 ± 0.0001	0.23
0.01% Cu/ZnO	0.0394 ± 0.0002	0.0373 ± 0.0002	0.21
1% Cu/ZnO	0.0393 ± 0.0003	0.0378 ± 0.0002	0.15
0.01% Ag/ZnO	0.0393 ± 0.0003	0.0385 ± 0.0002	0.08
1% Ag/ZnO	0.0396 ± 0.0003	0.0393 ± 0.0003	0.03

## Conclusion

- A simple and feasible Sol–Gel modified method was used in preparing functional cellulose sheets has been demonstrated, involving the use of Ag/ZnO and Cu/ZnO nanopigments for coating surfaces.
- The hexagonal structure of ZnO nanoparticles, decorated with small amounts of spherical Ag and Cu, was confirmed through TEM, HRTEM, XPS, and XRD analyses.
- The study examined the impact of varying dopant concentrations of Ag and Cu nanoparticles in composite coatings on antimicrobial properties. Even after 25 years of aging, pure ZnO nanoparticles retained antimicrobial activity. Increasing the concentrations of Ag and Cu significantly enhanced the effectiveness against *S. aureus*. Specifically, coatings with 1% Ag/ZnO and Cu/ZnO achieved bacterial inhibition rates of 92.2% and 62.1%, respectively, compared to 86.4% and 34.3% for 0.01% concentrations.
- Ag/ZnO and Cu/ZnO modified nanopigments improved the mechanical properties of the coated paper. Cu/ZnO has the largest enhancement effect.
- The use of Ag/ZnO nanoparticles had no significant effect on optical properties, despite Ag’s gray-dark color.
- However, Cu/ZnO reduced the brightness and whiteness of the coated paper without affecting opacity.
- Balancing enhanced mechanical properties with high antibacterial activity is crucial. Ag/ZnO and Cu/ZnO modified nanopigments are ideal for improving paper quality by offering both high mechanical strength and effective bacterial infection inhibition.
- ZnO, Ag/ZnO, and Cu/ZnO nanoparticles interact with the *S. aureus* MurA enzyme and cell wall synthesis genes, suggesting their potential as antimicrobial agents targeting bacterial cell wall integrity and viability.
- Water contact angle values for paper coated with Cu/ZnO are higher in comparison to those coated with Ag/ZnO at the same concentration.

## Data Availability

The datasets used and/or analyzed during the current study are available from the corresponding author on reasonable request.
